# How do medical students learn in an online community diagnostics program?

**DOI:** 10.1186/s12909-023-04007-8

**Published:** 2023-01-10

**Authors:** Junji Haruta, Takayuki Ando, Seitaro Fujishima

**Affiliations:** 1 Medical Education Center, School of Medicine, Keio University, 35 Shinanomachi, Shinjuku-Ku, Tokyo, 160-8582 Japan; 2 Center for General Medicine Education, School of Medicine, Keio University, Tokyo, Japan

**Keywords:** Realist approach, Community diagnosis, Learning mechanism, Community-oriented, Evaluation, Learning outcomes

## Abstract

**Background:**

The need to engage medical students in understanding the social and environmental determinants of health in disparate communities is increasing. However, previous reviews have noted the limited community diagnosis programs and program evaluation. Given the feasibility of the programs, it is expected to be widely available online. Therefore, this study used a realist approach to identify learning patterns through an online community diagnosis program, namely context (C), mechanism (M), and outcomes (O) patterns.

**Methods:**

A 2-week general medicine clinical practice program was conducted for 4^th^- and 5^th^-year medical students at a medical university in Japan. The program included a one-hour zoom-based lecture, feedback for students on their presentations on community diagnosis, and a structural report on community diagnosis. We developed the program based on variation theory, which views discernment and variation in situations having time, space, and social dimensions as core learning. The students' reflections on their learning through the program were thematically analyzed through CMO perspectives. The realist approach used in the online diagnosis program evaluation allows us to explore, test, and refine what mechanisms work under what conditions (context) and with what interventions (including opportunities and resources), from which we can describe iteratively explainable results.

**Results:**

First, the medical students, who spent most of their time in the limited residential areas they lived in, discovered the characteristics of their own community by discovery learning and comparison among peers. Second, they increased their intrinsic interest in the community by discerning specific issues in their familiar community through community diagnosis. Third, they valued community diagnosis by identifying relationships between local data on health issues under their learning responsibility. Fourth, they become more flexible in their thinking and created new knowledge that would fit the local community, and their reflection on themselves was encouraged.

**Conclusion:**

In this online community diagnosis program, medical students learned about the community through four types of learning patterns. Medical students may develop an understanding of community with interest using variation theory as a program development perspective and cognitive flexibility theory surrounding the essential ambiguity and abstraction of community.

**Supplementary Information:**

The online version contains supplementary material available at 10.1186/s12909-023-04007-8.

## Background

Health outcomes are affected not only by biomedical factors such as genetics and environmental exposures, but also by powerful social factors, specifically the social determinants of health (SDH), as defined by socioeconomic, environmental, and healthcare status [1, 2]. In particular, disparities in health based on socioeconomic factors are increasing not only among individuals, but also among communities. As one response, community health assessments (CHAs) are considered meaningful in reflecting on SDH in the community [3]. CHAs can assist in prioritizing issues in the health of a local community, and acquiring the ability to create and implement them is an essential skill for healthcare professionals and students. In medical education, where many specialists focus exclusively on biological issues, developing such a curriculum for medical students is needed because medical educators have a responsibility to train physicians to advocate for the most vulnerable members of society [4, 5].

Some papers have reported that understanding the determinants of population health and developing community health assessment skills enable physicians to practice effectively in diverse and underserved areas [6–9]. Many medical schools in the United States have established curricula focused on local community and population health, cultural sensitivity, health disparities, and socioeconomic determinants of health, and a framework to enhance prevention and population health content in the training of clinical health professionals has been developed [10–12]. Despite these efforts, however, a report released in 2015 examining the state of community health education stated that there is still room for improvement in education on population health [13].

In Japan, education in community health, such as community diagnosis, is still insufficient [14]. The WHO defines community diagnosis as “a quantitative and qualitative description of the health of citizens and the factors which influence their health. It identifies problems, proposes areas for improvement and stimulates action [15]” Community diagnosis programs are effective in rural and suburban settings and for pre-clinical students and those studying for master’s degree, who have relatively more time [16, 17]. However, few reports have investigated whether clinical practice for medical students promotes effective community diagnosis. In addition, while community diagnosis programs may provide medical students some knowledge and skills [16], few reports have described how these programs facilitate learning by medical students. Owing to COVID-19, many medical universities switched much of their training in clinical practice to online delivery in 2020 [18], and it has become increasingly difficult to implement community diagnosis programs that emphasize fieldwork to learn about community health. Given the increasing density of the medical education curriculum and need for cost-effectiveness, we anticipate that the online delivery of community diagnosis programs will meet the needs of society and education [19]. Identifying the process by which medical students learn about community health in such community diagnosis programs is expected to help faculty teach and medical students learn the abstract concept of community. To date, however, previous reviews have noted limited dissemination of SDH programs, a paucity of selected articles detailing the programs, and a lack of established tools and methods for evaluating learners and programs [4].

The effective realist approach is considered suitable for evaluating complex programs such as learning SDH. It is based on investigation of how, under what conditions, and with what context-based dynamism and realities such programs work. Here, to identify innovative patterns of the learning process, we evaluated the context in which the online community diagnosis program was implemented (C: Context), by what mechanism it worked (M: Mechanism), and what kinds of learning outcomes (O: Outcome) were acquired using it.

## Methods

### Study design

The realist approach seeks to answer the question of "what works, why, and how, in what circumstances," with realism as the paradigm that lies between positivism and social constructivism [20, 21]. In this approach, we first establish a working hypothesis we wish to investigate in the context of an educational program. Next, from purposefully collected quantitative or qualitative data, we explore, test, and refine what mechanisms work under what conditions (context) and with what interventions (including opportunities or resources), from which we can describe explainable outcomes in an iterative fashion. This is described by the formula *context* + *mechanism* = *outcome* (following prior literature, the components are described as CMOs, an acronym for Context-Mechanism-Outcome) [22]. The key to validating CMOs is in the consistency and integration of the CMOs refined from the working hypotheses on the one hand with the data collected based on those hypotheses on the other [23]. This analysis process is described in detail in the Realist approach section below.

### Curriculum of medical students in Japan

We briefly describe here the medical education curriculum in Japan to aid understanding of the learning context [24]. In Japan, students enter medical school after graduating from high school. The curriculum is presented in a six-year program that begins in April. In the first and second years, students begin lectures and practical training in basic medicine such as anatomy and physiology. In the third and fourth years, they study clinical medicine, including internal medicine and surgery. Students must pass a CBT (Computer-Based Testing) and OSCE (Objective Structured Clinical Examination) prior to clinical practice in order to be awarded the title of "student doctor" and be eligible for clinical practice. Clinical practice at the university in this study is conducted from January of the fourth year to November of the sixth year, for a total of 72 weeks. JH and colleagues in other universities previously developed a community-based medical education (CBME) curriculum within clinical practice that involves 16–19 students on rotations every four weeks, all spending one to two weeks in clinics or small hospitals to learn SDH in local communities [25]. This type of program is generally delivered as part of the general medicine component of clinical practice during the fourth or fifth year at a few Japanese universities. In the present study, we conducted a program evaluation focused on online community diagnosis in student-friendly hometowns at X university, which differs from prior literature [25].

### General medicine in clinical practice

The general medicine in clinical practice component at X university was previously implemented from January of the fourth year to December of the fifth year. Clinical rotations consisted of 5–6 students every 2 weeks. Due to the corona virus epidemic, we changed to a full online clinical rotation from February to May 2020. Although clinical rotations at the university hospital resumed in August 2020, some online rotations remained. We therefore developed a community diagnosis program to make medical students aware of community health issues through online training in general medicine. Delivery of this online component and this study began in August 2020. JH is a general practice physician, has earned a PhD, and has published multiple qualitative studies, including studies involving realist approaches; TA is a general practice physician with 6 years of experience working in community hospitals and clinics, including qualitative research using reports; SF has been a physician for 40 years, has a PhD, and is program director of a general practice department; and JH is involved in education in collaboration with a general medicine department and has various opportunities to communicate with the other authors.

#### Learning theory

Community-based medical education has used experiential learning, communities of practice, and situated learning as theoretical frameworks [25]. However, few learning theories clearly explain the reality of the learning experience of medical students when learning about their own community [16]. Therefore, using variation theory, we considered core learning as discernment and variation in situations having time, space and social dimensions [26, 27]. We defined the learning about a community as “the important identification of things that occur under interaction between the learner and the learning object; and the variations that arise in this interaction are what take the essential characteristics of things into thought based on variation theory.” This helped in elucidating the learning experience of medical students.

#### Learning objectives

Two learning objectives were set.

- Understand the purpose and methods of community diagnosis.
- Identify local community characteristics and analyze community health issues specific to the local community.

#### Program schedule

During a one-hour zoom-based lecture on the first day of clinical practice, the significance of community diagnosis was presented, together with specific examples. Students could select their own area of residence or an area they were familiar with, such as their hometown, for community diagnosis. After one week, the medical students presented the community diagnosis they had researched, and received feedback from peers and an author (JH). On the final day of the two-week program, each student presented a structural report on community diagnosis which included their own feedback-based assumptions or opinions, and submitted a revised report. To avoid one researcher (JH) bias, TA interviewed the medical students on the last day of the program to obtain their feedback on their learning, in place of JH.

#### Student assessment

Summative assessment was implemented by the first author based on a rubric. This assessment included evidence from the midterm and final presentations, and structured reports of the community diagnosis. This rubric was shared with the medical students on the first day. The author participated in observations about the students' presentations on the midterm and final days of the program; their listening attitude during their peers' presentations; and how they responded to feedback from peers and the author. Formative assessment was also conducted throughout the midterm and final day presentations. Feedback was provided mainly on the validity of what was examined in the community diagnosis, the logic of their explanation of the issues in the community, and the relevance and feasibility of the action plan.

### Setting and study participants

The setting of this study was the Department of General Medicine at X University in Tokyo, Japan, from August 2020 to December 2021. The academic year of Japanese universities begins in April. Our university starts clinical practice in January of the 4th year, and the subsequent 24-month period until December of the 5th year is the basic clinical practice period. The rate of female medical students in Japan is reported to be low; in 2018, only 21.9% of physicians were women [28]. Study participants included 4th- and 5th-year medical students who participated in a general medicine clinical clerkship in the basic clinical practice program.

### Realist approach

The analytical method was carried out according to the four steps of Pawson [22]. First, we formulated a working hypothesis that we aimed to investigate in the program. Working hypotheses about the program were formulated using deductive and inductive methods [22]. On this basis, using purposefully collected, mainly qualitative data, we explored, tested, and refined what mechanisms worked, under what conditions (context), with what interventions (including opportunities or resources), and from which explainable outcomes could be described and verified iteratively. This heuristic is to remind us to think of realistic evaluation in terms of constructs, and not as formulas, as in mathematics.

#### Generating a working hypothesis about the components of CMOs

Context is the condition under which the program is introduced, and is related to the mechanism, which considers "under what circumstances" the program will work. Mechanism is the process of how an individual interprets and acts on an intervention. Outcomes are how a series of outcomes result in an effect or change. We began by hypothesizing about potential mechanisms. Astbury and Leeuw reported that a clear distinction should be made between mechanisms and program interventions [29]. For example, an outcome might be an increase in the learners’ knowledge or readiness resulting from some mechanism arising from some educational intervention. In other words, the mechanism is an explanatory model that can be described in relation to multiple context and educational intervention variables. Dalkin et al. used the formula presented by Pawson et al., *Context* + *Mechanism* = *Outcome* [22]. With regard to the mechanism of the program's intervention and the mechanism as an inference caused by the program, Dalkin et al. proposed a method of describing the *context* + *mechanism (intervention) → mechanism (inference)* = *outcome* [30]. With this representation, mechanisms are explained by both concrete facts and interpretive inferences. The contexts in which program interventions are introduced vary, as do the emerged patterns of mechanisms (inferences) and activated outcomes. Thus, CMOs describe multiple patterns of how the various components of a program harmonize and integrate. Here, to describe these patterns, we utilized the observational evaluation and community diagnostic structural reports submitted by the students on the final day of the program.

#### Observation and verification

The working hypothesis was tested by collecting data on CMOs. At the end of the fiscal year, program evaluation was conducted based on the medical students' verbal and written reflections and reports. The authors confirmed whether a series of CMOs could comprehensively explain the learning patterns of the community diagnostic program as an evaluation. When testing for consistency and integration, we did not focus solely on a single outcome, but examined whether CMOs as learning patterns of community diagnosis under various combinations of learner outcomes with contexts and mechanisms allowed readers to transfer the findings to other settings.

#### CMOs clarification

This process of validation and refinement resulted in a pattern of CMOs, namely a series of mechanisms and learner outcomes which could explain the complex learning process based on several contexts. Continued validation and refinement of the CMO patterns was conducted using data collected over the two years [31].

### Ethical approval and consent to participate

This study was approved by the Ethics Committee of Keio University (approval number: 20211157), and was performed in accordance with the Declaration of Helsinki. All participants were given the opportunity to opt out in the web-page of the medical education center at Keio University. Informed consent was obtained from all participants.

## Results

### Working hypothesis

The study participants included 4^th^- and 5^th^-year medical students who completed a two-week program in general medicine from August 2020 to December 2021. The 171 medical students in this study undertook the online community diagnosis program from August 2020 to December 2021. 32 were female.

To incorporate the essential features of community diagnosis, lectures and feedback were given that provided an overall understanding and specific examples. The first day's lecture presented students with a general overview and specific examples of individual, family, community, and social systems theory related to community diagnosis. Feedback in the midterm and final presentations was given in a way that made students aware of the variation theory (Table 1). Utilizing the variations generated in the process, we asked some questions with the goal of broadening or shifting the individual student’s focus. We also verbalized and explained the community differences that emerged through comparison of the communities presented by the students.

**Table 1:** Field note data of the students and faculty's interactions in the community diagnosis program sessions

	**Medical student expression data**	**Faculty’s assessment of medical students**	**Feedback strategy**	**Data: specific assessments (A) and feedback (F)**
1	This town has a high suicide rate and I would like to come up with a plan to reduce it	The community’s judgement is biased and prejudiced, and a simple plan is presented based on that judgment	Facilitate comparison of stratified data and exploration of their relationship to the context	(A) He could not understand community context (F) Which age group has more suicides than Japan as a whole? What factors might be behind that?
2	X area has few medical facilities, so it would be good to increase the number of medical facilities	Judgments are made with a view to the subject area only	Present perspectives in relation to the surroundings	(A) Not capturing issues in relation to neighboring wards with many large hospitals (F) What are the neighboring wards like? What is the status of medical facilities in those wards?"
3	The aging rate is not so high in this area, and there are enough hospitals and other facilities, so there is no need to think about medical care issues in this area	Time frame not captured	Present time horizons	(A) You are trying to make a decision based on only one part of the recent data (F) Why isn't this town aging as well as some other cities?" In 5–10 years, when we are all doctors, how will the demographics of this town have changed?"
4	I thought that if the price of medical checkups were reduced by about 1,000 yen (approx. $7), the rate of checkups would increase	Misalignment of group characteristics and action plans	Reconfirm target population characteristics	(A) Failure to capture the barriers to accessing health screenings for hard-working generations (F) Do you think everyone's father or mother would go for a health checkup if it cost ¥1,000 less?"
5	I thought the average household income was low in this district, and therefore happiness was also declining	Seeing cause and effect from one's own natural perspective	Encourage reflection on their assumptions	(A) Stereotypical perspective (F) I wonder if the average household income is really lower in towns with lower levels of happiness?" Bhutan has a high level of happiness, but what about household income?"
6	If they're healthy on the physical exam, why not give them a financial incentive?	Expects to benefit from information and resource inputs only	Consider implications for Behavioral Science Interventions	(A) Not assuming the disadvantages of monetary intervention as an incentive, and viewing the art of motivating people only in terms of major interventions (F) Why do you take a medical checkup? To get money? Doesn't that change your sense of purpose?" It's hard for someone without symptoms to get a physical, but do you know the term “nudge” in behavioral economics?

In addition, a structural report (supplemental file 1) was prepared so that medical students could follow the process of community diagnosis. The report was structured into five steps. In Step 1, the report categorized community information and provided hints for information retrieval. In Step 2, a section titled “Listening to residents' opinions” presented examples of how to clarify local opinions through interviews and fieldwork, as well as how to search real estate websites for information on local residents' opinions of the community. Step 3 described the process of conducting interviews and walking around the community based on a hypothesis developed from the information gathered above. Examples included "Which generations of people walk around town?" and “How many supermarkets are there? What kind of people use them, and at what times?". Although the state of emergency due to COVID-19 had been declared over, it was difficult to casually conduct interviews, so many students had to talk to family and friends in their hometowns online or by other means. They also conducted their fieldwork mainly online. All students researched their familiar hometowns and communities well; many students had hometowns in the metropolitan vicinity, but about 20 student participants had hometowns in other parts of the country and had diverse community backgrounds.

Step 4 helps participants identify the community’s strengths and weaknesses. Finally, in Step 5, participants are guided in planning the implementation of activities to solve the community’s issues, from the perspective of feasibility, cost-effectiveness, and sustainability.

The working hypotheses were explored from August to December 2020 during our implementation of a community diagnosis program to raise awareness of community residents’ health issues and their relationship to local factors. As a result, we asked the following as working hypotheses: “How does the context (Context) influence medical students in an online community diagnosis program; what is the process of organizing the relationship between local community factors and the online program interventions (Mechanism); and how do they verbalize the community and the role of medicine and physicians (Outcome)?”.

### Testing and refining the hypothesis of CMOs

All training was conducted and the reports were evaluated by the first author (JH). First, data analysis was conducted by the author (JH), and thematic analysis was conducted on the basis of feedback and observations during the practical training, and reports prepared by the students [32]. Second, as triangulation to establish a working hypothesis and validate the learning pattern as CMOs, the other authors (TA and SF) confirmed the data and interpretation as the effect of the program. Third, based on the analysis, JH developed the pattens of CMOs. The pattern of CMOs was further refined with TA and SF. The following table shows the results of the study.

### Patterns of configuration of CMOs (Tables 2 and 3)

Four patterns of configuration of CMOs emerged from the above process. The following series of context-mechanism-outcomes is described in Table 2. In the interaction between the context of some medical students and the context of online program, multiple mechanisms functioned to result in outcomes that promoted motivation and reflection in the learners. The mechanism (intervention) describes the learner's experience as extracted from the data, and the mechanism (reasoning) describes the learning process as inferred from the data. Table 2 helps the reader understand the CMO patterns by explaining each pattern, and Table 3 describes representative data that we interpreted.

1. Reflections on the familiar local community elicited by integrating discovery learning and comparison.
2. Intrinsic intellectual curiosity generated by discerning familiar community issues through community diagnosis
3. Value-associated interest triggered by identifying the relationships between health problems and community data regarding learning responsibility
4. Reflection on oneself directed by stimulating cognitive flexibility to identify complex relationships

**Table 2:** Four patterns of configuration of CMOs (Context, Mechanisms, and Outcomes)

	**Context (Students)**	**Context (Online program)**	**Mechanisms (interventions)**	**Mechanisms (reasoning)**	**Outcomes**
1	Medical students who grew up with limited contact with the community	Each student can share and present the data they have researched via Zoom with the students in their group	Share the results of the student’s own community diagnosis and the specific results of the student’s team members' community diagnosis	Significance learning integrating discovery learning and comparison organizers	Facilitate reflection on their own familiar local community
2	Medical students with little interest in community health issues	Students themselves select the local community in which they will study	Detailed analysis of familiar local issues from multiple perspectives based on the structured five-step report	Promoting understanding of essential content that shows the connection between local community’s issues and health problems	Enhanced intrinsic intellectual curiosity in community diagnosis
3	Medical students who tend to be closed off and syncretistic within an inner group of their peers	Online situation where different local communities’ diagnoses are examined for each individual, and a personal screen is assigned over Zoom	Hypothesis generation from data and questions from faculty to recognize the logic, and their reflection on preconceptions of individuals on learning responsibility	Deriving abductions by identifying relationships among community data	Attracting value-associated interest in community diagnostics
4	Medical students who believe that a formulaic approach is the only solution	Online practice with time to spare	Trial and error in information seeking to examine the relationship between local community background and health issues	Fostering cognitive flexibility to identify the relationships among complex and changeable elements within the whole	Reflection on themselves in relating knowledge structure with community issues. Students’ perspectives changed from a unidirectional perspective to a more interactive view

**Table 3:** Representative data extracted from CMOs based on the medical students' final reports

1	Reflections on the familiar local own community elicited by integrating discovery learning and comparison	Student A: "Recently, the manager of a new grocery store in my neighborhood was always chatting with a local family as if they were friends, and I was very interested in the fact that the area that had not been bustling before became lively every day after that grocery store opened, regardless of the social class of people, so I turned my attention to the community during this training. It was a very meaningful experience for me personally because I was able to understand academically how the manager's actions impacted and contributed to the community." Student B: "I have rarely thought about the health issues in the town where I grew up, and it made me realize that the reason these issues exist may be because I, as a member of the local youth, was not interested in them." Student C: "In preparing for my presentation, I was able to sense that the characteristics of X city that I investigated (far from Tokyo) were different from those of the cities of the other presenters, and that these characteristics could be successfully reconciled by consideration based on regional background."
2	Intrinsic intellectual curiosity generated by discerning familiar community issues through community diagnosis	Student D: "Interestingly, I noticed that I have almost no network with the elderly in my neighborhood. Two houses away and you don't even know who lives there anymore, which I think is an indication that community-based communities are more sparse than they used to be." Student E: "I felt that the problem in urban areas can be summarized as how to encourage patients who do not belong to the community to improve their health checkup rates and lifestyle-related diseases in a highly viable and sustainable manner. In particular, I felt that this is a problem unique to urban areas, where the hurdles to joining a community alone are high due to the high number of people moving in and out of the community. I could recognize it as an urban problem where we live"
3	Value-associated interests triggered by identifying relationships between health problems and community data on learning responsibility	Student F: "In my previous training, I have dealt with each patient's disease by asking about his/her medical history and life history, but in this community diagnosis, in order to examine the health issues of the people in the region, in addition to the direct health checkup rate and prevalence rate, I also examined the geographical characteristics, industry, and health of the region, which seemingly have no direct relation to health. It was very interesting to see how indirectly local issues came to the surface by examining factors such as population, connections, infrastructure, etc., and I learned the importance of viewing things from a variety of perspectives." Student G "My friend mentioned neighborhood associations as a way to deal with "isolation," and in my thinking stage, I thought about preventing "isolation" by successfully matching the parent–child generation, which is common in Y town, with the elderly, but I thought that the neighborhood association connection was not quite in line with the times and abandoned further thinking. However, when you mentioned the importance of making the best use of the original Japanese neighborhood association and redefining the definition of that connection, I was glad to hear that you had thought of that."
4	Reflection on oneself directed by stimulating cognitive flexibility to identify complex relationships	Student H. "I learned that it is easy to link one factor to an outcome when considering the regional nature of a disease, for example, but it is not easy to consider whether there are other factors and which one has the greater influence, so I learned that we should take as broad a view as possible when considering the regional nature of a disease." Student I: "When I thought about a concrete action plan, I realized that I would need the cooperation of quite a few people when I thought about where and with whom I would need to cooperate. I don't know if I will ever be involved in social prescribing as a physician in the future, but if I do, I will need to cooperate with various people in the community, so I hope I can build good relationships with them on a regular basis."

#### Reflections on the familiar local own community elicited by integrating discovery learning and comparison

Some medical students grew up with limited contact with the community. Through the online program, each medical student shared and presented the data they had researched about their local community with a group of medical students via Zoom to learn about the different community diagnoses of other medical students. As a result, students were able to reflect on their own local community by identifying similarities and differences through discovery learning and comparisons among peers. Students confirmed the uniqueness of the local community they examined, and verbalized integrated and meaningful findings about the community.

#### Intrinsic intellectual curiosity generated by discerning familiar community issues through community diagnosis

Some medical students had little interest in community issues because their learning primarily involved medicine-centered knowledge. Thus, to motivate these students, we let them pick up the local community area they lived in or were familiar with. Thus, they were able to analyze familiar community issues with interest in details from multiple perspectives based on the five-step structured report. The intrinsic intellectual curiosity of community diagnosis could be enhanced by promoting understanding of essential content through discernment of the issues of a community with which they are familiar.

#### Value-associated interest triggered by identifying the relationships between health problems and community data regarding learning responsibility

Because the clinical training of medical students is conducted by rotation on a peer group basis, some could be considered free riders within their groups who lacked a sense of personal responsibility. *(A student said, “I am online more and more after the corona pandemic, and I don't get to go to clinical sites as often, and I am not allowed to do it as a medical student. The clinical training is mainly group work, so if participants don't try to do something on their own, they are allowed to do nothing” Some students agreed with this. -observation data).* On the other hand, use of an online setting, such as each student sharing a screen view of their report via Zoom, can provide each individual with learning responsibility to share their own local communities’ diagnoses. In this process, they can implement abductive reasoning—that is, the logical process of applying the reasoning that makes the most sense regarding the phenomenon—by identifying the relationships among the collected data, and answer the questions from faculty to recognize the logic and preconceptions by themselves. Their interest could be activated through the process of deriving abductions; they were able to find value in community diagnostics; and they were able to implement more refined abductions through the learning process.

#### Reflection on oneself directed by stimulating cognitive flexibility to identify complex relationships

Medical students who are accustomed to multiple choice questions with fixed answers tend to believe in formulaic approaches which involve only one solution. (A few students asked JH the question, “*Why should we have to think for ourselves about local community issues that are not necessary for clinicians, like a high school student's free summer research project (with no right answer)?” -observation data)* An online environment with relatively free time and space for thinking about unanswered questions through trials and errors can allow students to develop the cognitive flexibility required to learn about relationships among complex and changeable elements within a whole, such as community diagnosis. They can reflect on themselves in their relating the local knowledge structure with complex community issues. Through the learning process, some students' perspectives changed dramatically, namely from a perspective of unidirectionality and causality to a more interactive view or topography- and history-related perspective according on the context of the times.

## Discussion

This study is one of only a few educational investigations into community diagnosis and identification of the learning patterns of medical students. In a UK report, community diagnosis was conducted in third-year medical students in small group tutorial teaching, supported by a GP tutor [16]. Similarly to the present study, the students in that study appreciated the value of investigating their community and commented on the opportunity to consider the broader determinants of health. However, few papers have investigated the learning patterns of students in community diagnosis [16].

GPs, in discovering, relativizing, and identifying community values and needs, seek to identify conditions that meet community expectations, and satisfy those values [33]. Similar to GPs, medical students reflected on their familiar communities through discovery learning and comparison through online community diagnosis. Discovery learning has been shown to enhance transfer of learning and increase trainees' positive attitudes [34]. However, some have argued that it places unnecessary stress on trainees due to the risk of inaccurate content and the cognitive load of struggling [35, 36]. Given that the medical students in this study who did not do well in discovery learning expressed frustration, it may be better to develop opportunities for the provision of feedback from other perspectives, such as peers and community members. On the other hand, while community-based medical education (CBME) frameworks for understanding communities suggest opportunities for small group activities ranging from simple to complex learning, they often remain limited to general educational principles such as experiential learning, transformative learning, and feedback [37]. Even if CBME is implemented based on the principles of these educational theories, the impact on medical students' learning may be less than that expected if faculty lack understanding of education in this particular practice field [38]. In particular, physicians working in primary care settings are often entrusted with the education of medical students without adequate preparation in undergraduate medical education [39]. A paper showed that it is difficult for such GPs to encourage medical students to understand the value of community [40]. This present study revealed that teaching community diagnosis in an online environment, where the few medical education professionals who are proficient in CBME have the freedom of time and space, can help overcome these current challenges by collaborating with GPs in a community.

In addition, motivation for learning was raised by taking multiple perspectives on the community and identifying the breadth and depth of these perspectives. This, according to variation theory, is the core of learning: discernment and variation. “There is no learning without discernment. And there is no discernment without variation.” Without discernment, there can be no learning [41]. How we experience a situation depends on how we discern the important features of this situation [41–43]. Thus, it is clear that the theory of variation is consistent with the learning process of community diagnosis, which integrates complex elements. To learn about the relationships among complex elements, the cognitive flexibility theory is an auxiliary line of learning mechanisms [44]. This theory, as presented by Spiro and colleagues, emphasizes that the nature of learning in complex and ill-structured domains is about preparing people to select, adapt, and combine knowledge and experience in new ways to deal with situations which differ to those they have previously encountered. One principle of this theory involves providing multiple representations of contents as learning activities. Our present community diagnosis program makes this possible by the sharing of community diagnoses among colleagues in a group. A community diagnosis in which each student attempts an abduction using local community data, rather than a didactic lecture, would allow medical students to select, adapt, and combine knowledge to solve local community issues. The cognitive flexibility theory may prove foundational in supporting medical student learning about unstructured domains, such as community diagnosis. Such flexible thinking may have been promoted through comparison with other local communities and with other learners' perspectives, and through various methodologies [45].

Healthcare providers have a responsibility to show students how to engage with the socio-economic realities of health inequalities [46, 47]. The specific data from the community, coupled with the students' familiarity with the community and their personal learning responsibilities, made them realize the value of community diagnosis. We would like to suggest reconfiguration of the current medical education model and the need to consider medicine not only in terms of biomedical evidence, but also in terms of the contemporary socioeconomical realities of patients and lay people [48]. In the redevelopment process, we hope that medical faculties and students as healthcare advocates can embrace the fact that we are part of the community's resources and have a responsibility to shape them.

Several limitations of our study warrant mention. First, data collection was based on a hypothesis and theory developed by the authors, and thus a degree of researcher bias may be present. To mitigate this, three authors critically examined the validity of these data and refined them via iteration three times over two fiscal years. Furthermore, the CMOs were described to explain the context, allowing readers to transfer the findings to other settings, albeit that the verification of CMOs developed in this study in other settings is required.

With regard to implementation, the learning patterns in gaining an understanding of community may provide clues for the development of medical curricula that require opportunities to teach social and behavioral science in medicine, insofar as the findings of this study can be considered transferable to other contexts. Additionally, the online community diagnosis learning patterns have significant international impact as a theoretical framework. Additionally, when faculty understands variation theory as a perspective of program development, the cognitive flexibility theory that surround the essentially ambiguous and abstract nature of community, medical students may deepen their understanding of community with interest. Discovery learning, variation theory, and cognitive flexibility theory may work in community diagnosis even though the effectiveness of face-to-face community diagnosis also needs to be verified. Although selection bias must be taken into account—given that this is a single university study—we hope that prescribing these CMOs may help the development and implementation of community diagnostic programs. This can be validated in other settings by evaluating the program with an awareness of whether a series of CMOs occurs as learning patterns. In addition, since this program was conducted as an online community diagnosis program, it can be implemented without considering geographical characteristics, such as those specific to Japan or to urban areas.

## Conclusion

A realist approach in online community diagnosis revealed four learning patterns of medical students with regard to the community. Through a community diagnosis program, medical students can deepen their understanding of communities by intrinsic intellectual curiosity using discovery learning, variation theory, and cognitive flexibility theory as program development perspectives on the essential ambiguity and abstraction of communities. These findings will have a major impact in informing medical educators who deal with community-related topics about the potential of community diagnosis.

## Supplementary Information

**Additional file 1.** Community diagnosis report.

## Data Availability

All data analyzed during the current study are available from the corresponding author on reasonable request.
