# Supplementary material for: How do medical students learn in an online community diagnostics program?
Source: BMC Med Educ. 2023 Jan 10;23:15. doi: 10.1186/s12909-023-04007-8 (PMC9830126; doi:10.1186/s12909-023-04007-8)
Supplement: Supplementary file 1 — Additional file 1. Community diagnosis report. [file 12909_2023_4007_MOESM1_ESM.docx]

Community Diagnosis Report

**Step 1 Explore local community issues** (collect and organize existing community information and provided hints for information retrieval)

1. **Objective indicators, statistical data related to the health of residents, status of infrastructure and institutions, etc.**

　Examples: population structure, demographics, life expectancy, healthy life expectancy, mortality statistics, disease statistics, medical cost statistics, long-term care related statistics, etc.

　　　Public services, medical institutions, nursing and welfare facilities, care services, schools, kindergartens and nurseries, transportation, stores, etc.

　　　Community activities, volunteer activities, sports activities, hobby club activities, senior citizen club activities, etc.

　　　Industrial structure, places to work, tourism resources, etc. ・・・ How do these compare to the rest of the country?

<Advice & Supplemental Explanation

　Include the source (URL) so that it can be referenced later.

　Consider not only statistical data, but also infrastructure and institutions.

　Do not focus only on shortages and issues, but also write down what has been sufficiently accomplished (fulfilled).

　For all tables below, additional rows may be added.

| (data) item | Survey source (URL) and summary of results |
| --- | --- |
|  |  |
|  |  |

*If space is insufficient, please increase the number of rows.

1. **Observe and listen to the community (gather information from many sources, not just objective data)**

Collect photos of the community, health and welfare staff, patients and users, shoppers, townspeople, and other voices online!

<Explanation> Community diagnosis requires not only objective data, but also real voices and subjective data.
 Gather information online while imagining what you would ask locals (and who you might ask) in your chosen region.

e.g. Information on the Internet (objective information, subjective information of the sender, etc.)
 Virtual inspection using aerial photos on mapping sites, etc.

| Theme | What was said | The person (position) who was speaking |
| --- | --- | --- |
|  |  |  |
|  |  |  |

*If space is insufficient, please increase the number of rows.

**Step 2** Listening to residents' opinions; prepare for fieldwork (community survey and site visit)

**Step 3** Conduct interviews and city walking fieldwork (interviews and site visits)

Based on the data from Step 1, formulate a hypothesis that may be related to local issues and describe the results of interviews with staff at the training facility and local residents (especially resident organizations, community leaders, etc.), as well as the results of a community walk in the area surrounding the training facility.

Hypothesis 1.

| Who should be interviewed and what should be asked in order to test the hypothesis?  Where should we focus our attention on walking around the city to test our hypothesis? | Results and Comments from the Listening and Research |
| --- | --- |
|  |  |
|  |  |

*If space is insufficient, please increase the number of rows.

**Step 4 Community assessment (analyze information and identify health issues)**

Fill in the table below by thinking about the health problems that can be expected in this area and the local problems that may cause health problems (what is needed and what is lacking) from the perspective of "so that people can continue to live healthily here”.

1. **Synthesize the information gathered regarding the hypotheses and organize the strengths and weaknesses (problems) of the site.**

| Strengths of the area (bullet points) | Problems in the area (bullet points) |
| --- | --- |
|  |  |

1. **Indicate in one sentence the local issue to be taken up this time**

Describe for what (purpose), to whom (target), toward what (goal), and what will be done (intervention).

|  |
| --- |

**Step 5 Action Plan (develop and implement a plan of action to solve local issues)**

<Tip>

　Who should we collaborate with?

Comprehensive community support centers, nursing care departments, social welfare councils, chambers of commerce and industry, residents' mutual aid organizations, community leaders, etc.

　How can we improve the situation? (do we need to educate the public and hold workshops (human resource development))?

　How much budget is likely to be needed?

Also, be aware of the following points and make a plan with the intention of making a realistic proposal to the local mayor.

| Proposals to improve problem areas by leveraging strengths (high priority and feasibility) |
| --- |

**[Reflection on community diagnosis].**

|  |
| --- |
